# Effect of Maturation on the Chemical Composition, Nutritional Value, Biological Activities In Vitro and Volatile Organic Compounds in the Coconut Meat of Wenye No. 3 Variety

**DOI:** 10.3390/foods15173136

**Published:** 2026-09-03

**Authors:** Xiaoyan Liu, Yufeng Zhang, Qi Gao, Jintao Kan, Fei Song

**Affiliations:** 1 Hainan Engineering Center of Coconut Further Processing, Coconut Research Institute of Chinese Academy of Tropical Agricultural Sciences, Wenchang 571339, China; liusmile18@163.com (X.L.);; 2 College of Modern Agriculture and Bioengineering, Shangluo University, Shangluo 726000, China; 3 College of Tropical Crops, Yunnan Agricultural University, Pu’er 665099, China

**Keywords:** coconut meat, maturity, proximate composition, antioxidant activity, α-amylase inhibition, volatile organic compounds

## Abstract

Coconut meat (solid endosperm) is a valuable oilseed product with significant nutritional and functional properties, yet the quality changes during maturation remain unclear. This study investigated the effects of maturity on nutrients, antioxidant and α-amylase inhibitory activities, and volatile organic compounds in coconut meat of the Wenye No. 3 variety at 8, 10, and 12 months after pollination (named CM-8, CM-10 and CM-12, respectively). Results showed that moisture content decreased from 81.15% to 61.73%, while fat (8.61% to 17.53%), protein (0.60% to 1.29%), and total soluble sugars (2.13% to 4.84%) increased from CM-8 to CM-12. Total phenolic content declined with maturity, whereas ABTS scavenging activity was significantly higher at CM-12 than at CM-10, and hydroxyl radical scavenging activity was similarly elevated at CM-10 and CM-12. α-Amylase inhibitory activity peaked at CM-10 (20.4%). Phosphorus, magnesium, zinc, and copper peaked at CM-12. Lauric acid predominated at all stages, with unsaturated fatty acids transiently increasing at CM-10. GC-IMS identified 31 volatile compounds, with esters and alcohols dominating in CM-8 and CM-10, while aldehydes accumulated in CM-12. These findings provide theoretical guidance for staged harvesting and graded utilization of coconut in the processing industry.

## 1. Introduction

Coconut (*Cocos nucifera* L.) is a distinctive oilseed crop of significant economic and nutritional value in tropical regions, occupying a central position in the tropical fruit and vegetable processing industry [1,2]. The coconut meat, also called as solid endosperm, is the core part of the entire coconut fruit and has the highest value. Rich in high-quality plant protein, characteristic medium- and short-chain fatty acids, dietary fiber, and various other nutritionally active substances [3], it serves as the key raw material for producing coconut milk, cold-pressed coconut oil, shredded coconut, and active coconut peptides, and drives the core output value of the coconut processing industry [4]. The lauric acid account for nearly 50% of the characteristic lipids in coconut meat. Coconut protein is also a high-quality plant protein source with a balanced amino acid composition and demonstrated antioxidant and antihypertensive activities [5]. Driven by its unique nutritional and functional characteristics, coconut meat and its processed derivatives have gained growing market share in the global functional food industry.

Fruit maturity stage largely determines the nutritional composition, functional properties and flavor profiles of coconut meat. For instance, the phenolic composition of Pistacia lentiscus fruits varies significantly across ripening stages [6], and the harvest maturity of kiwifruit determines its postharvest storage performance [7]. Thus, clarifying the dynamic changes in basic nutrients, antioxidant activity and volatile organic substances of coconut meat at different developmental stages can support the establishment of scientific maturity grading standards, reduce batch quality differences caused by mixed harvesting, and promote standardized and high-value utilization of coconut resources. However, existing studies mainly focus on the physicochemical characteristics and volatile compounds of tender coconut water harvested at 6–8 months after pollination [8,9]. Comprehensive research tracking the continuous variation of nutrients, antioxidant capacity and volatile organic compounds in coconut meat during key maturation periods is still scarce [10]. This research gap coincides with the common industrial problem of mixed harvesting of 8-month semi-mature to 12-month fully mature fruits without unified grading standards, which ultimately causes unstable quality of processed coconut products and restricts the development of high-end deep-processing industries.

Among available coconut varieties, dwarf cultivars exhibit superior fresh consumed and higher yield per unit performance compared with traditional tall coconuts, featuring early fruiting, stable high yield and uniform fruit texture [11,12]. Wenye No. 3 is a typical dwarf cultivar bred from the introduced “Malayan Red Dwarf” by the Coconut Research Institute of CATAS, and was officially released in 2007. It has been widely promoted across Hainan Province, where its early bearing, high yield, and excellent nut quality have generated significant economic returns.

Our previous study investigated the chemical components, nutritional value, and volatile organic compounds of coconut water from the Hainan Tall variety at identical maturity stages [13], revealing that all these parameters exhibited distinct changes with advancing maturity. For example, the total phenolic content peaked at 10 months, while ABTS and hydroxyl radical scavenging activities were highest at 8 months and declined thereafter. However, the coconut meat of the dwarf cultivar Wenye No. 3, which serves as the primary raw material for coconut processing, has not been systematically characterized across these key maturity stages. Given the distinct physiological functions and chemical compositions of liquid and solid endosperms, as well as the genetic differences between tall and dwarf cultivars, the quality evolution patterns of coconut meat during maturation may differ substantially from those previously observed in coconut water.

Therefore, in this study, coconut meat of the widely cultivated dwarf variety Wenye No. 3 in Hainan Province was collected at three key maturity stages (8, 10, and 12 months after pollination). The dynamic changes in proximate composition, nutritional value, antioxidant and α-amylase inhibitory activities, and volatile organic compounds were systematically evaluated, with the aim of elucidating the quality evolution of coconut meat during maturation and providing a scientific basis for staged harvesting and graded utilization.

## 2. Materials and Methods

### 2.1. Plant Material Preparation

**Figure 1:**
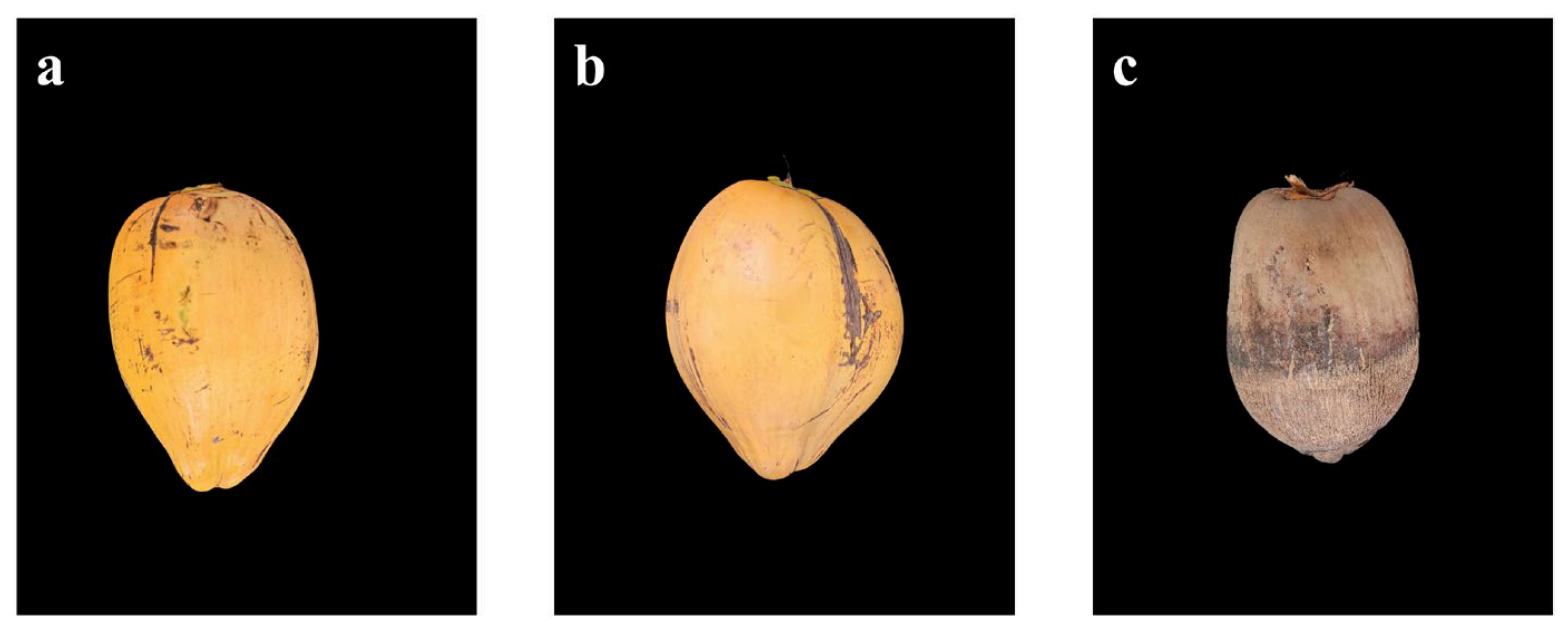
Appearance of Wenye No. 3 coconut fruits at 8 (**a**), 10 (**b**), and 12 (**c**) months after pollination.

The test material consisted of Cocos nucifera var. Wenye 3 collected from the National Tropical Palm Crop Germplasm Resource Nursery in Wenchang City (19°32′42.9″ N, 110°47′35.6″ E, Hainan, China). The coconuts were harvested in batches at different stages of development (8, 10, and 12 months after pollination), with three individual fruits collected at each stage as biological replicates (*n* = 3). Only fruits with intact, undamaged husks, uniform shape, and no signs of pest or disease infestation were selected for subsequent experiments (Figure 1). After harvest, coconut fruits were transported to the laboratory within 2 h. The fruits were dissected with sterilized knives, and coconut meat was carefully excised. The coconut meat was snap-frozen in liquid nitrogen for 10 s, pulverized, aliquoted into sealed bags, and stored at −80 °C for further analysis. Composite samples were not prepared; each fruit was analyzed separately to account for biological variation. The coconut meat samples at different stages of maturity (8, 10, and 12 months after pollination) were named CM-8, CM-10 and CM-12, respectively, and results are expressed as mean ± SD of the three biological replicates.

### 2.2. Determination of Proximate Components

#### 2.2.1. Moisture, Ash, Fat, and Protein Content

The proximate composition of coconut meat, including moisture, ash, fat and protein, was determined following the standard AOAC methods (935.29, 938.08, 920.39 and 2001.11, respectively).

#### 2.2.2. Total Soluble Sugar Content

The total soluble sugars were determined using a previously reported method [13], with slight modifications. Approximately 2 g of freshly ground coconut meat was mixed with 40 mL distilled water and subjected to ultrasonic extraction for 30 min. The resultant extract was centrifuged at 5000 r/min for 30 min, and the supernatant was collected for subsequent determination. For analysis, 8 μL of the supernatant was diluted with 992 μL water, then mixed with 4 mL anthrone reagent. The mixture was heated in a boiling water bath for 10 min, and the absorbance was measured at 620 nm to quantify total soluble sugars.

#### 2.2.3. Crude Fibre Content

The crude fiber content in coconut meat was determined according to a previously described method [14]. Briefly, 20 g crushed fresh coconut meat was boiled gently in 200 mL of boiling 1.25% sulfuric acid for 30 min (shaken every 5 min). The residue was washed with hot water until neutral, then re-boiled in 200 mL of boiling 1.25% potassium hydroxide solution for 30 min. After filtration and hot water washing, the residue was suction-filtered in a crucible, washed with hot water, ethanol and diethyl ether in turn, and dried at 105 °C to constant mass for weighing.

### 2.3. Total Phenolics Content

Accurately weigh 2 g fresh coconut meat, extract with 40 mL 60% ethanol via ultrasonication for 30 min using an ultrasonic cleaner (KQ-500DE, Kunshan Ultrasonic Instruments Co., Ltd., Kunshan, China). The extract is centrifuged at 5000 r/min for 30 min, and collect the supernatant for use in the subsequent total phenolics content, antioxidant, and α-amylase inhibitory activity assays. Absorbance measurements were recorded using a UV-visible spectrophotometer (UV-2600, Shimadzu Corporation, Kyoto, Japan).

Total phenolics were determined according to the Folin phenol method, as previously described (with minor modifications) [15]. Briefly, pipette 1 mL of the supernatant, then add 2.5 mL of Folin-Ciocalteu reagent and 2.5 mL of 7.5% sodium carbonate solution sequentially. Shake well and keep in the dark for 2 h. Subsequently, add 4 mL of distilled water and let stand at room temperature for 1 h. Finally, the absorbance of the mixture was measured at 765 nm. Total phenolics content (TPC) was expressed as mg gallic acid equivalents (GAE) per gram of fresh weight (mg GAE/g), using gallic acid as the standard.

### 2.4. Assessment of Antioxidant and α-Amylase Inhibitory Activities In Vitro

#### 2.4.1. ABTS Radical Scavenging Activity

In the subsequent experiments to determine antioxidant and enzyme-inhibitory activities, the supernatant was diluted with 60% ethanol at a 1:4 volume ratio to prepare the sample solutions.

The ABTS working solution was prepared according to the reported method [16], with minor modifications. Mix 0.5 mL sample solution with 4.0 mL ABTS working solution, incubate at 30 °C for 10 min, then record absorbance A_1_ at 734 nm. Replace sample solution with 60% ethanol to obtain A_0_, and replace ABTS working solution with anhydrous ethanol to obtain A_2_. The ABTS radical scavenging rate is calculated using the following formula:

(1) $$\mathrm{Radical}\;\mathrm{scavenging}\;\mathrm{rate}\;(\%)=\frac{A_{0}-A_{1}+A_{2}}{A_{0}}\times 100$$

#### 2.4.2. DPPH Radical Scavenging Activity

The DPPH working solution preparation and determination were based on the reported method [17]. Briefly, the sample solution (0.5 mL) and 0.1 mM DPPH working solution (2 mL) were mixed in a test tube. Next, the mixture was kept in the dark for 30 min. The absorbance of the mixture was measured at 517 nm (A_1_), while the blank control (A_0_), with the sample replaced by 60% ethanol and the sample control (A_2_), with DPPH replaced by methanol) were measured under the same conditions. Similarly, the DPPH radical scavenging rate of the sample solution was calculated using Formula (1).

#### 2.4.3. Hydroxyl Radical Scavenging Activity

The reagents and experimental procedures used to determine the hydroxyl radical scavenging activity of the sample solution were based on previous methods, with minor adjustments [18]. Mix 1 mL of sample solution, 6 mM FeSO_4_, 6 mM salicylic acid-ethanol and 6 mM H_2_O_2_, incubate at 37 °C for 30 min, and record absorbance A_1_ at 510 nm. Replace sample solution with 60% ethanol to obtain A_0_, and replace salicylic acid-ethanol solution with 60% ethanol to obtain A_2_. The hydroxyl radical scavenging rate was calculated by Formula (1).

#### 2.4.4. Ferrous Ion Chelating Ability

The ferrous ion chelating ability assay was performed according to the reported method [19] with minor optimizations. Briefly, 1 mL of 0.1 mM FeSO_4_ solution, 1 mL of 0.2 mM ferrozine solution and 1 mL of sample solution were sequentially added into a test tube. After thorough mixing, the mixture was incubated at room temperature for 10 min, and the absorbance was recorded as A_1_ at 562 nm. The absorbance of blank control (A_0_) was obtained by replacing the sample solution with 60% ethanol, while the absorbance of sample blank (A_2_) was measured by substituting FeSO_4_ and ferrozine solutions with distilled water. The chelating ability was calculated according to Formula (2).

(2) $$\mathrm{Ferrous}\;\mathrm{ion}\;\mathrm{chelating}\;\mathrm{rate}\;(\%)=\frac{A_{0}-A_{1}+A_{2}}{A_{0}}\times 100$$

#### 2.4.5. α-Amylase Inhibitory Activity

The α-amylase inhibitory activity was carried out following the reported method [20] with minor optimizations. Mix 500 μL sample solution with 500 μL α-amylase solution and incubate at 37 °C for 10 min. Then add 500 μL 1% (*w*/*w*) soluble starch solution and incubate at 37 °C for another 10 min. The reaction was terminated by adding 1 mL DNS reagent, followed by heating in a boiling water bath for 10 min. After cooling the mixture to room temperature, 10 mL distilled water was added for dilution and mixing, and the absorbance was measured at 540 nm and recorded as A_1_. Three control groups were set simultaneously to eliminate system interference. The absorbance of sample blank (A_2_) was measured by replacing α-amylase with buffer; the absorbance of test control (A_3_) was obtained by substituting sample solution with 60% ethanol, while the absorbance of reagent blank (A_0_) was measured by replacing sample solution with 60% ethanol and α-amylase with buffer. The α-amylase inhibitory activity was calculated according to Formula (3).

(3) $$\mathrm{Inhibitory}\;\mathrm{activity}\;(\%)=\left[1-\frac{A_{1}-A_{2}}{{A_{3}-A}_{0}}\right]\times 100$$

### 2.5. Nutritional Value Evaluation

#### 2.5.1. Amino Acid Composition

Weigh an appropriate amount of the ground coconut meat sample and place it in a hydrolysis tube. Add 10 mL of 6 mol/L hydrochloric acid solution, purge the air from the tube with nitrogen, seal it, and hydrolyze at 110 °C for 22 h. After hydrolysis is complete, cool to room temperature, dilute to a final volume of 25 mL with water, and filter under reduced pressure for later use. Accurately pipette 1 mL of the filtrate and evaporate to dryness under reduced pressure; redissolve the residue in 2 mL of pure water and evaporate to dryness again. Thoroughly dissolve the resulting solid in 1.0 mL of a pH 2.2, 0.067 mol/L sodium citrate-hydrochloric acid buffer solution. Filter the solution through a 0.22 μm water-based filter membrane to obtain the test solution. The amino acid composition was determined using an L-8900 automatic amino acid analyzer equipped with a cation-exchange analytical column. The instrumental detection parameters and calculation methods for mass proportions of individual amino acids were referred to the reported method [13].

#### 2.5.2. Fatty Acid Composition

Fatty acid composition of fresh coconut meat was analyzed via lipid methylation following prior studies [21]. Briefly, the clarified methylated solutions were subjected to fatty acid composition analysis using an Agilent 7890A gas chromatograph (Agilent Technologies, Wilmington, DE, USA) equipped with a Supelco SP-2560 capillary column (100 m × 0.25 mm, film thickness 0.20 μm) and a flame ionization detector (FID). GC operating conditions were set as follows: injection volume, 1.0 μL; nitrogen as carrier gas; split ratio, 100:1; inlet temperature, 270 °C; detector temperature, 280 °C. The oven temperature program was implemented in multiple stages: an initial hold at 100 °C for 13 min, followed by heating to 180 °C at 10 °C/min and holding for 6 min; the temperature was then increased to 200 °C at 1 °C/min and maintained for 20 min; finally, the oven was ramped up to 230 °C at 4 °C and held for 10.5 min. Individual fatty acids were identified by matching their retention times with those of external standards. The relative content of each fatty acid was calculated as the percentage of its peak area relative to the total peak area of all detected fatty acids.

#### 2.5.3. Mineral Elements

Mineral elements in coconut meat were determined by optimizing an established method [22]. Briefly, 0.25 g of dried and crushed coconut meat was accurately weighed and mixed with 6 mL of nitric acid, followed by pre-digestion at room temperature for 1 h in a fume hood. Subsequently, 2 mL of 30% hydrogen peroxide was added, and the mixture was subjected to gradient temperature-programmed digestion using a MARS 5 microwave digestion system (CEM Corporation, Matthews, NC, USA). The digestion procedure was set as follows: heating to 120 °C within 8 min and holding for 2 min, raising the temperature to 160 °C within 5 min and maintaining for 5 min, and further heating to 180 °C within 5 min with a constant-temperature digestion for 15 min. After cooling to room temperature, the digested solution was diluted to a constant volume of 100 mL with ultrapure water and mixed thoroughly for subsequent determination. An Agilent 7700X inductively coupled plasma mass spectrometer (Agilent Technologies, Santa Clara, CA, USA) was used for elemental analysis with the following operating parameters: radio frequency power of 1600 W, carrier gas flow rate of 1.0 L/min, peristaltic pump speed of 0.1 r/s, and spray chamber temperature of 2 °C. Throughout the experiment, the oxide formation index and double-charge ion ratio were strictly controlled at 0.45% and 1.01%, respectively.

### 2.6. Profiling of Volatile Organic Compounds (VOCs)

The identification of VOC in coconut meat was performed based on an existing method [23] with adjustments made to some parameters. Approximately 3.0 g of each coconut meat sample was placed inside a 20 mL headspace vial with 1 μL of 2-methyl-3-heptanone (0.1 g/L) as the internal standard. The samples were incubated at a constant temperature of 60 °C for 20 min, followed by adsorption and enrichment of headspace volatile components using pre-treated extraction fibers. After adsorption, the extraction fiber was inserted into the injector of the FlavourSpec^®^ gas chromatography-ion mobility spectrometry (GC-IMS) instrument (G.A.S., Dortmund, Germany) for thermal desorption at 85 °C, so that the volatile compounds captured by the fiber were released for instrumental detection. Parameters for ion mobility spectrometry (IMS): the drift tube temperature was set to 45 °C; high-purity nitrogen (99.99%) was adopted as the drift gas at a flow rate of 150 mL/min. Parameters for gas chromatography (GC): the chromatographic column was maintained at a constant temperature of 60 °C; nitrogen was used as carrier gas with a gradient flow program: constant at 2.0 mL/min within 0–2 min, linearly increased to 10 mL/min from 2 min to 10 min, and further linearly ramped up to 100 mL/min during 10–20 min. Volatile compounds were quantified using an internal standard method, and the content of each compound was calculated based on the peak area ratio to the internal standard and expressed as μg/kg on a fresh weight basis. Data acquisition and processing were performed using VOCal software (version 3.0, G.A.S. mbH, Dortmund, Germany).

### 2.7. Statistical Analysis

Data analyses were performed using SPSS 26.0 (IBM Corp., Armonk, NY, USA), and results were expressed as mean ± standard deviation. One-way analysis of variance (ANOVA) with Duncan’s multiple range test was applied for multiple statistical comparisons. A *p*-value < 0.05 was defined as statistically significant. All experiments were performed in triplicate for reliable and reproducible results.

## 3. Results and Discussion

### 3.1. Proximate Composition Analysis

Table 1 presents the proximate composition of coconut meat at various developmental stages. From CM-8 to CM-12, moisture content decreased sharply from 81.15% to 61.73%, while fat, protein, and total soluble sugars increased significantly, reaching 17.53%, 1.29%, and 4.84% at CM-12, respectively. This inverse relationship between water loss and nutrient accumulation is a distinctive feature of coconut meat development and is consistent with previously published research reports [24]. The progressive dehydration of the coconut meat creates a concentrated matrix that favors lipid and protein deposition, as water is gradually replaced by storage reserves [25].

Notably, the total soluble sugar content remained relatively stable between CM-8 (2.13%) and CM-10 (2.26%) but surged to 4.84% at CM-12. In developing coconut meat, sucrose and reducing sugars serve as both energy sources and carbon skeletons for fatty acid biosynthesis, and the concurrent increase in fat content from 8.61% to 17.53% during this period does indicate active lipogenic activity [26]. However, the nearly twofold increase in total soluble sugars from CM-8 (2.13%) to CM-12 (4.84%) suggests that sugar accumulation exceeds the immediate demands of fatty acid synthesis. From a seed biology perspective, this may reflect a physiological preparation for germination, as the haustorium, which develops inside the nut upon germination, requires readily available soluble sugars from the endosperm to support embryo development [27]. Meanwhile, crude fiber content was approximately 1.5-fold higher at CM-12 (13.36%) than at CM-8 (8.85%). It decreased slightly from CM-8 to CM-10 and then increased markedly at CM-12. The initial decrease may be associated with pectin and hemicellulose degradation during early maturation, while the significant increase at CM-12 reflects cell wall thickening and lignification, which contributes to the firmer texture characteristic of mature coconut meat. This structural hardening is important for industrial processing, as it influences shredding, milling, and extraction efficiencies.

### 3.2. Phenolic Content Analysis

**Figure 2:**
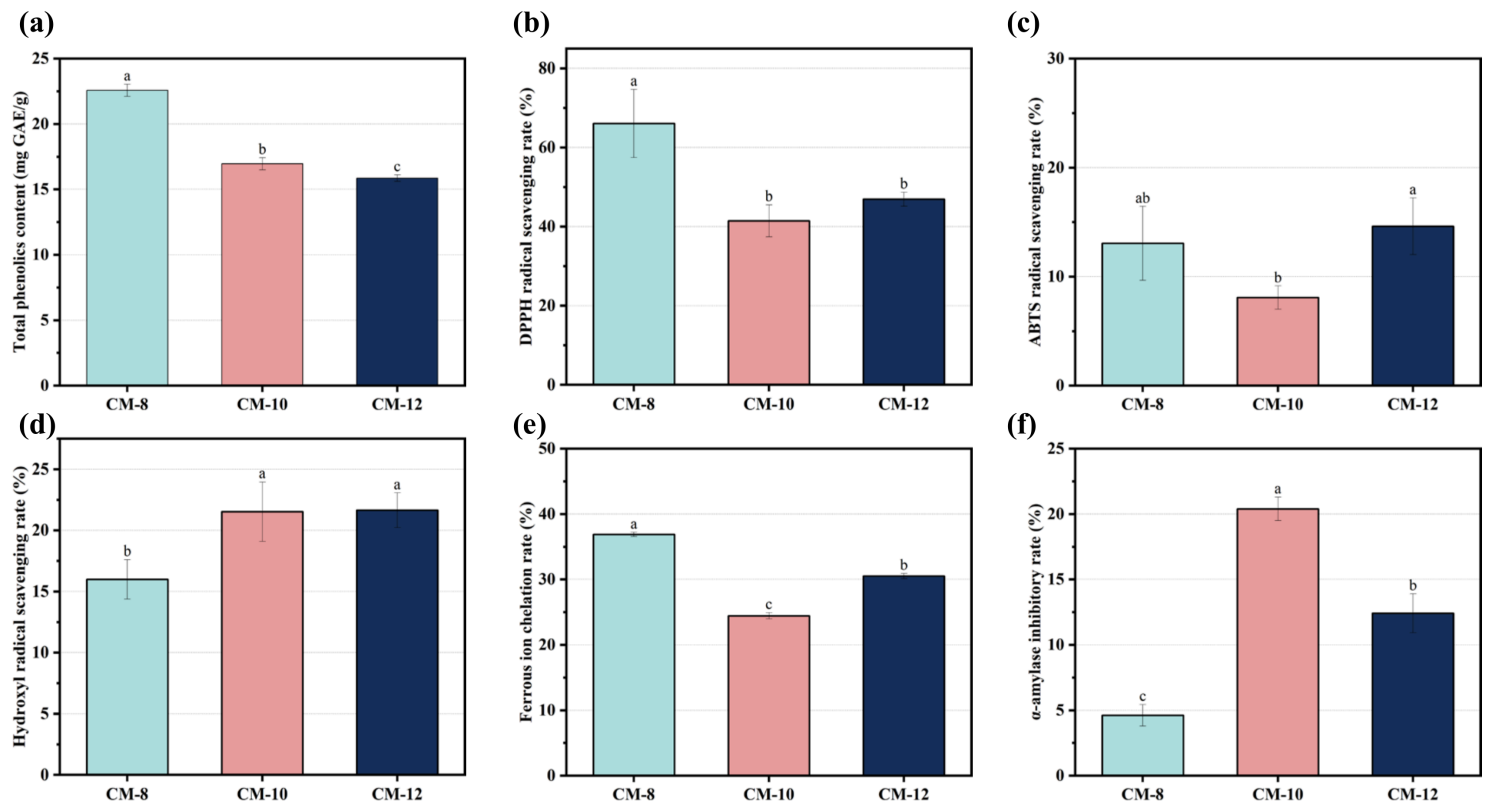
Changes in total phenolic content (**a**); DPPH (**b**); ABTS (**c**); Hydroxyl radical (**d**) scavenging activities; Ferrous ion chelating ability (**e**); and α-amylase inhibitory activity (**f**) of coconut meat at different maturity stages. Values marked with different lowercase letters (a–c) within the same row are significantly different at *p* < 0.05.

As shown in Figure 2a, the total phenolic content (TPC) of Wenye No. 3 coconut meat exhibited a significant decreasing trend with advancing maturity, decreasing from approximately 22.57 mg GAE/g at CM-8 to 16.96 mg GAE/g at CM-10, and further to 15.86 mg GAE/g at CM-12 (*p* < 0.05). This trend is consistent with previous observations in coconut and other oilseed crops, where phenolic compounds typically accumulate at higher levels in immature tissues and gradually decline as the organ shifts toward storage reserve deposition [10]. From a processing perspective, the distinct TPC levels at different maturity stages determine their optimal applications. Coconut meat harvested at 8 months, with its higher phenolic content, is well suited for the production of natural antioxidant extracts for nutraceutical, cosmetic, and food preservation applications, offering a natural alternative to synthetic antioxidants. In contrast, 12-month mature coconut meat, despite its lower TPC, is the preferred raw material for traditional coconut oil production. The reduced phenolic level in fully mature meat may help minimize enzymatic browning during the initial milling and refining stages of oil processing, as polyphenol oxidases catalyze the oxidation of phenolics into dark pigments. However, phenolic compounds are valuable natural antioxidants that protect oils from oxidative deterioration during storage. Therefore, selecting appropriate maturity stages based on specific processing requirements is essential. For instance, coconut meat harvested at slightly earlier maturity stages, with higher phenolic content, is more suitable for producing products where phenolic bioactivity is desired, such as coconut milk powder and desiccated coconut, whereas fully mature meat remains preferable for conventional oil production where lighter color is prioritized [28]. Additionally, TPC values (22.57–15.86 mg GAE/g) of Wenye No. 3 coconut meat are comparable to those reported by Mahayothee et al. [16], who found that TPC of coconut meat from Thai aromatic coconuts varied with maturity and declined after 190 days after pollination. Jiang et al. [10] also reported significantly higher TPC in tender coconut meat than in mature coconut meat in a dwarf cultivar, which is consistent with our finding that TPC decreased from CM-8 to CM-12.

### 3.3. Analysis of Antioxidant Activity

The antioxidant capacity of coconut meat at different stages of ripeness was evaluated using four assay methods: the ABTS and DPPH radical scavenging assays, the hydroxyl radical scavenging assay, and the ferrous ion chelating capacity assay. The results indicate that the antioxidant activity of coconut meat is related to the coconut’s degree of ripeness. As shown in Figure 2b, DPPH radical scavenging activity was highest at CM-8 (66.08%) and significantly lower than that of CM-10 (41.47%) and CM-12 (46.94%). This decline is consistent with the downward trend in total phenolic content, suggesting that phenolic compounds may be the primary source of the coconut pulp extract’s hydrogen-donating capacity [8]. A similar trend has been observed in Thai aromatic coconut meat, where DPPH activity declined with advancing maturity [16]. In contrast, ABTS radical scavenging activity in Figure 2c exhibited a different trend: CM-12 was the highest (14.62%), followed by CM-8 (13.05%), with CM-10 showing the lowest (8.08%). The discrepancy between the DPPH and ABTS results can be attributed to the different mechanisms of radical scavenging between the two assays. DPPH is dissolved in an alcoholic medium and primarily reflects the hydrogen-donating capacity of hydrophilic antioxidants. In contrast, the ABTS assay is conducted in a water-organic biphasic system and can simultaneously detect both hydrophilic and lipophilic antioxidants [29]. The lower DPPH activity at CM-12 is consistent with the lower phenolic content, likely because phenolic compounds are the primary hydrophilic radical scavengers. In contrast, the ABTS assay may include contributions from certain lipophilic antioxidants (such as tocopherol and tocotrienol), which accumulate in fully mature coconut meat as lipid content increases. This trend differs from previous reports on coconut meat, where ABTS activity generally declined with advancing maturity [16], suggesting that the accumulation of lipophilic antioxidants in fully mature Wenye No. 3 may offset the loss of phenolic compounds in the ABTS system.

As can be observed from Figure 2d, the hydroxyl radical scavenging activity increased from 15.99% at CM-8 to 21.53% at CM-10 and 21.65% at CM-12. The activity of CM-10 and CM-12 showed no significant difference between them, but both were significantly higher than that of CM-8 (*p* < 0.05). The ferrous ion chelating ability in Figure 2e was highest at CM-8 (36.89%), followed by CM-12 (30.53%), and lowest at CM-10 (24.45%), with significant differences among all three stages (*p* < 0.05). These variations in antioxidant activity across the four assays can be attributed to the different mechanisms of action involved, since certain antioxidants react rapidly, slowly, or remain inert toward specific radicals [30]. In summary, no single maturity stage shows the highest antioxidant activity across all assays. DPPH scavenging and ferrous ion chelating were highest at CM-8; ABTS scavenging was significantly higher at CM-12 than at CM-10, while CM-8 showed intermediate activity with no significant difference from either. Hydroxyl radical scavenging was similarly elevated at CM-10 and CM-12. Therefore, the optimal maturity depends on the specific antioxidant target for the intended application.

### 3.4. Analysis of Enzyme Inhibitory Activity

The α-amylase inhibitory activity of coconut meat extracts showed significant differences (*p* < 0.05) depending on the degree of ripeness. As shown in Figure 2f, the CM-10 exhibited the highest α-amylase inhibition rate, reaching 20.4%; CM-12 followed with an inhibition rate of 12.42%; CM-8 exhibited the lowest inhibitory activity, at only 4.62%. This trend differs markedly from the changes observed in total phenolic content during coconut meat maturation. Although CM-8 had the highest total phenolic content, its α-amylase inhibitory activity was at the lowest level, indicating that phenolic compounds are not the primary functional components responsible for the α-amylase inhibitory activity of coconut meat. Based on this, it is hypothesized that the α-amylase inhibitory activity of coconut meat may originate from other bioactive compounds, such as characteristic peptides, organic acids, or non-phenolic secondary metabolites [31], and that these active compounds tend to accumulate or increase in activity during the mid-to-late stages of coconut meat ripening.

CM-10 exhibited the optimal α-amylase inhibitory activity, indicating that coconut meat harvested at this stage of maturity may have the potential to mildly inhibit starch digestion and delay postprandial glucose release when consumed with starchy foods. However, compared to reported highly effective α-amylase inhibitors such as legume extracts and acarbose [32,33], the inhibitory capacity of coconut meat extracts is relatively weak. Further studies are needed to evaluate its efficacy through in vivo experiments and to explore its practical applicability as a functional food ingredient.

### 3.5. Amino Acid Composition Analysis

As can be observed from Table 2, the amino acid composition of coconut meat exhibited a pronounced increase with advancing maturity. Across all three stages, glutamic acid and arginine were the most abundant amino acids, while methionine and histidine were present at the lowest levels, consistent with the typical amino acid profile of coconut proteins reported previously [5]. The contents of both essential amino acids (EAA) and non-essential amino acids (NEAA) increased significantly (*p* < 0.05) with maturity. Total EAA nearly doubled from 5.41 mg/g at CM-8 to 10.46 mg/g at CM-12, and total NEAA increased from 11.86 mg/g to 22.20 mg/g during the same period. Despite the marked increase in absolute amino acid content, both the EAA/TAA (total amino acids) and EAA/NEAA ratios remained relatively stable across all three stages, ranging from 31.25% to 32.06% and from 0.46 to 0.47, respectively.

Among the essential amino acids at CM-12, leucine (2.14 mg/g), lysine (1.69 mg/g), and valine (1.70 mg/g) were the most abundant, while histidine (0.78 mg/g) and methionine (0.55 mg/g) were present at lower levels. Among non-essential amino acids, glutamic acid (6.66 mg/g) and arginine (5.30 mg/g) were the most predominant. Glutamic acid is a key contributor to umami taste and may enhance the savory flavor profile of mature coconut meat, while arginine serves as a precursor for nitric oxide synthesis and plays important roles in immune function and wound healing [27]. The substantial accumulation of these amino acids during late maturation contributes not only to improved nutritional value but also to flavor development in processed coconut products, particularly during thermal processing where amino acids participate in Maillard reactions to generate roasted and nutty aromas. From a nutritional perspective, the near-doubling of EAA content from CM-8 to CM-12 suggests that fully mature coconut meat provides substantially higher levels of essential amino acids, making it a more valuable ingredient for food fortification and protein supplementation, while the stable EAA/TAA and EAA/NEAA ratios indicate that the amino acid balance is maintained throughout maturation.

### 3.6. Fatty Acid Composition Analysis

As shown in Table 3, the fatty acid profile of coconut meat exhibited distinct changes with maturity, particularly in the proportion of saturated and unsaturated fatty acids. Lauric acid (C_12:0_) remained the predominant fatty acid across all stages [10], accounting for 51.22% at CM-8, 41.16% at CM-10, and 46.87% at CM-12, with significant differences among all three stages (*p* < 0.05). This dominance of lauric acid is a well-known signature of coconut oil and is responsible for many of its functional properties, including antimicrobial and immunomodulatory activities [34].

The most notable change occurred at CM-10, where oleic acid (C_18:1n9c_) appeared at 13.27% while lauric acid dropped to its lowest level. Consequently, total saturated fatty acids (SFA) decreased significantly (*p* < 0.05) from 98.75% at CM-8 to 85.19% at CM-10, while total unsaturated fatty acids (UFA) increased significantly from 1.25% to 14.81% (*p* < 0.05). This transient increase in UFA at CM-10 can be attributed to the progressive synthesis of oleic acid during the transition from CM-8 to CM-10. By CM-12, oleic acid declined to 5.66%, and SFA rebounded to 93.27%, indicating a shift toward the typical highly saturated profile characteristic of mature coconut oil. Jiang et al. [10] reported that as coconuts mature, the proportion of saturated fatty acids increases while unsaturated fatty acids decline, a shift likely associated with the upregulation of genes involved in MCFA biosynthesis. The elevated UFA content at CM-10 observed in our study may represent an intermediate stage in this maturation process, where unsaturated fatty acids temporarily accumulate before the final transition to saturated lipid storage.

From a nutritional and processing perspective, the high SFA content at CM-8 (98.75%) and CM-12 (93.27%) confers superior oxidative stability, making these stages more suitable for applications requiring long shelf life, such as frying oils and confectionery fats [25]. In contrast, CM-10, with its elevated UFA content (14.81%), may offer a more nutritionally balanced profile but with reduced oxidative stability. Furthermore, linoleic acid (C_18:2n6c_), an essential fatty acid, is present at all stages of ripeness, indicating that coconut meat has a certain nutritional value regardless of its ripeness. Overall, these findings demonstrate that maturity significantly influences the fatty acid composition of coconut meat, with CM-10 representing a distinctive stage with transiently higher unsaturation, while CM-8 and CM-12 provide the highly saturated profiles typical of commercial coconut products.

### 3.7. Mineral Elements Composition Analysis

As shown in Table 4, the content of various mineral elements in coconut meat exhibited varying patterns of change as the post-pollination age increased. Specifically, the phosphorus and magnesium contents were significantly higher at CM-12 (1049.08 mg/kg) than at CM-8 (617.79 mg/kg) (*p* < 0.05). Potassium content decreased significantly (*p* < 0.05) from 5097.32 mg/kg at CM-8 to 4277.12 mg/kg at CM-10, and then increased to 4753.91 mg/kg at CM-12, though it remained significantly lower than the CM-8 level (*p* < 0.05). Sodium content peaked at 210.84 mg/kg during the CM-10 stage, approximately five times that of the CM-8 and CM-12, with significant differences among all three stages (*p* < 0.05). The mineral composition of coconut meat has been characterized in several cultivars, with potassium being the most abundant macro-mineral [35,36]. However, cultivar-specific differences in mineral accumulation have been reported [35], and the transient sodium spike at CM-10 observed in the present study may reflect a cultivar-specific feature of Wenye No. 3. Calcium remained stable between CM-8 and CM-10 but decreased significantly at CM-12 (*p* < 0.05).

The concentrations of zinc, copper, and manganese all peaked at the CM-12 stage, showing significant differences (*p* < 0.05) compared to earlier stages. The trend in iron concentration was similar to that of potassium. The accumulation of zinc and copper in mature coconut meat is of great nutritional significance, as these elements serve as cofactors for Cu/Zn-SOD and are crucial for immune function and connective tissue metabolism [37]. The manganese content at the CM-12 stage is also worthy of attention, as it plays a crucial role in carbohydrate and lipid metabolism [14]. In terms of nutritional contribution, based on an estimated 100 g serving of fresh coconut meat, potassium and magnesium at all three stages exceeded the lower bounds of their RNI ranges, while phosphorus and copper only reached nutritionally valuable levels at CM-12. These findings suggest that coconut meat, particularly at CM-12, can serve as a good dietary source of phosphorus, magnesium, and copper. The high sodium content of CM-10 may have potential application value for sodium-supplemented formulations.

### 3.8. Analysis of Volatile Organic Compounds

**Figure 3:**
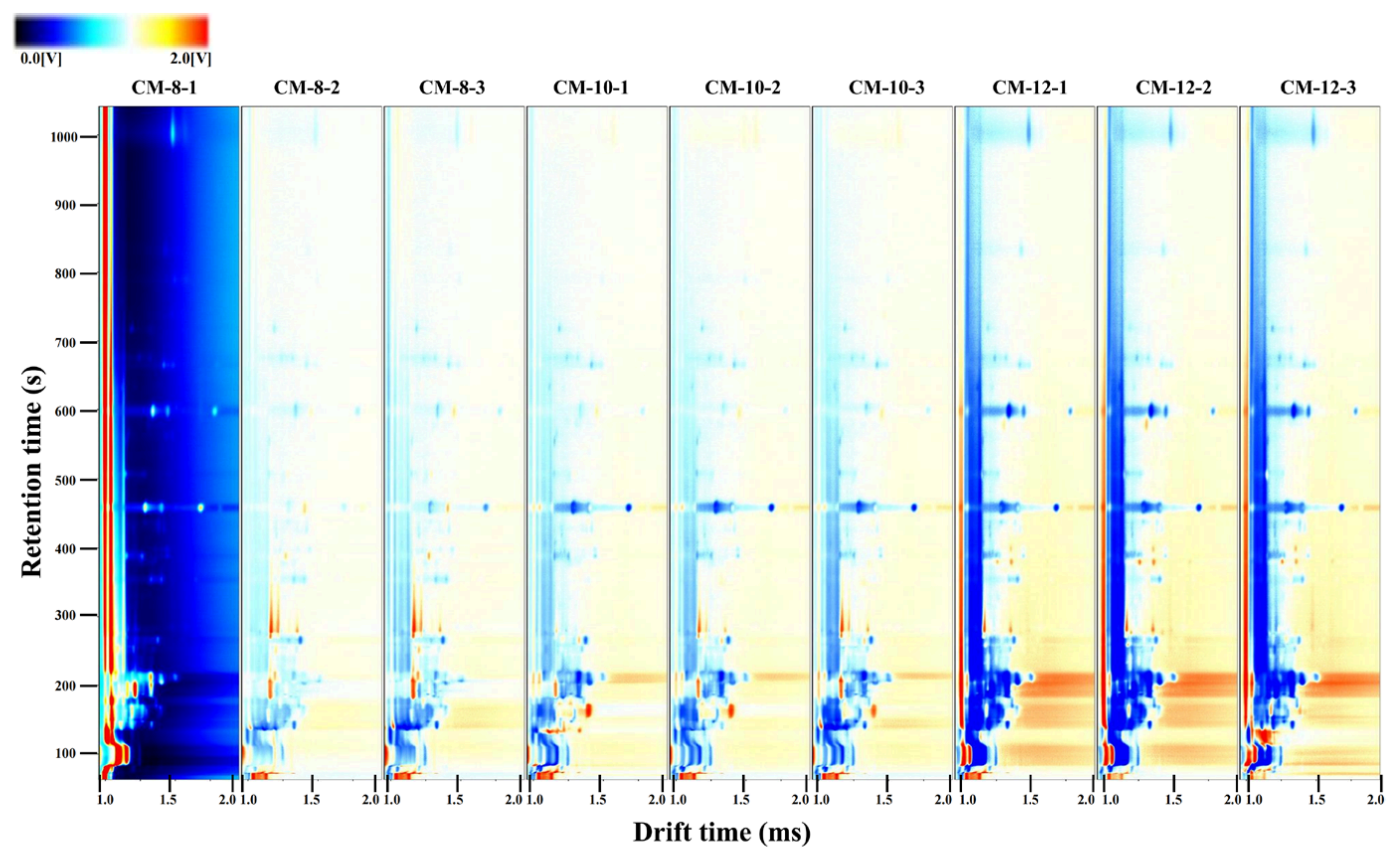
Differential GC-IMS spectra of volatile compounds in coconut meat at different maturity stages using CM-8-1 (the first replicate of the CM-8 sample) as reference. Red indicates higher concentration than CM-8-1, blue indicates lower concentration. Each dot represents a volatile compound, and color intensity reflects the magnitude of difference.

**Figure 4:**
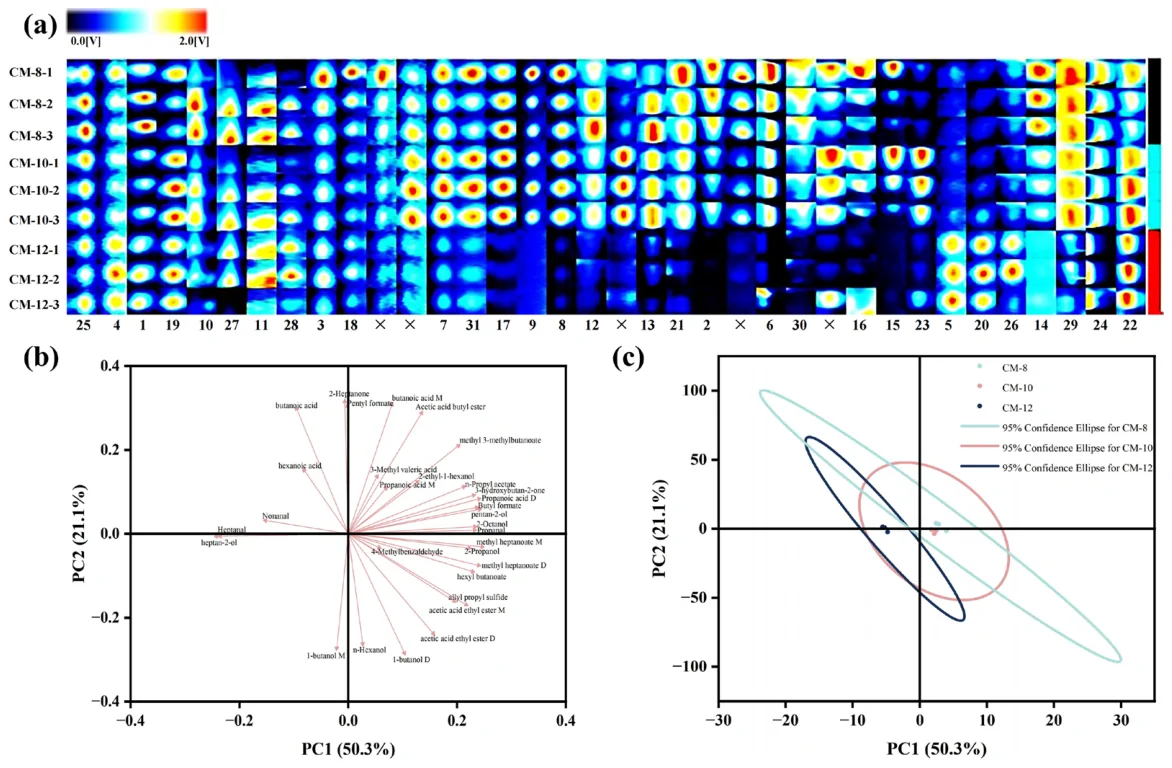
Volatile compound profiles of coconut meat at different maturity stages. Fingerprint (**a**); PCA loading plot (**b**); PCA score plot with 95% confidence ellipses (**c**). The numbers 1–31 in the bottom row of the fingerprint plot (**a**) correspond to the identified VOC listed in the first column of Table 5, whereas “×” denotes VOCs that could not be identified using the available databases.

The volatile organic compounds of coconut meat at three maturity stages were qualitatively and quantitatively characterized by HS-SPME-GC-IMS, with 31 compounds identified (Table 5). The differential spectra (Figure 3), using CM-8-1 (the first replicate of the CM-8 sample) as reference, visually illustrated the differences in volatile composition among the three stages-red indicating higher concentration than CM-8 and blue indicating lower concentration. The Gallery Plot fingerprint (Figure 4a) further displayed the signal intensity distribution of each compound across samples, with the red-boxed regions showing that CM-8 and CM-10 exhibited stronger signals in ester and small-chain alcohol regions, while CM-12 showed stronger signals for nonanal and heptanal. The PCA loading plot (Figure 4b) identified the key compounds contributing to group separation. Compounds located on the positive side of PC1, such as nonanal and heptanal, were more abundant in CM-12, serving as characteristic markers distinguishing fully mature samples from immature ones. In contrast, compounds on the negative side of PC1, such as *n*-propyl acetate, 2-propanol, propanal, and acetic acid ethyl ester, were more abundant in CM-8 and CM-10 and decreased substantially at full maturity. The PCA score plot (Figure 4c) revealed tight clustering within each group with non-overlapping 95% confidence ellipses, confirming significant maturity-dependent differences in volatile composition.

Among ketones, 2-heptanone was significantly lower (*p* < 0.05) at CM-10 than at CM-8 and CM-12, with no significant difference between the latter two. 3-hydroxybutan-2-one decreased consistently with significant differences among all three stages, nearly disappearing at CM-12 (*p* < 0.05).

Aldehydes showed accumulation at full maturity. Nonanal was significantly lower at CM-10 than at CM-12, but showed no significant difference from CM-8. Heptanal was significantly higher at CM-12 than at CM-8 and CM-10, with no significant difference between the latter two. These compounds are oxidative degradation products of oleic acid and linoleic acid, respectively [38], and their accumulation coincided with the substantial increase in total lipid content at CM-12. Among aldehydes, propanal was the most abundant volatile compound across all stages, decreasing sharply from 84.67 μg/kg at CM-8 to 22.24 μg/kg at CM-12, representing a reduction of approximately 74%. This marked decline suggests that short-chain aldehydes are progressively lost or metabolized during maturation. In contrast, 4-methylbenzaldehyde showed no significant changes across the three stages, remaining at relatively low levels (1.27–1.47 μg/kg). The accumulation of lipid-derived aldehydes at CM-12 indicates that fully mature coconut meat is better suited for thermal processing, where these aldehyde precursors can further develop roasted and nutty flavors [39].

Among esters, *n-*propyl acetate was the most abundant compound across all stages, remaining high at CM-8 and CM-10 with no significant difference between them (*p* > 0.05), but declining sharply at CM-12. Acetic acid ethyl ester (M and D) and methyl heptanoate showed no significant differences across stages. Hexyl butanoate reached its highest level at CM-10, significantly higher than at CM-8 and CM-12 (*p* < 0.05). The higher ester content at CM-8 and CM-10 suggests that these stages retain more fruity and floral aroma characteristics, making them suitable for fresh consumption or minimally processed products [13].

Alcohols displayed divergent trends. 1-Butanol M remained at higher levels at CM-10 and CM-12, both significantly higher than at CM-8, while 1-butanol D form increased significantly at CM-10 and then returned to CM-8 level at CM-12. 2-pentanol decreased consistently with significant differences among all three stages. 2-propanol remained high at CM-8 and CM-10 with no significant difference between them, but decreased significantly at CM-12 (*p* < 0.05).

Among acids, propanoic acid D decreased consistently with significant differences among all three stages (*p* < 0.05), while propanoic acid M, butanoic acid, and hexanoic acid showed no significant differences across stages. Methyl 3-methylbutanoate, which possesses fruity and sweet flavor profiles [39], was significantly lower at CM-12 than at CM-8 and CM-10, with no significant difference between the latter two.

In summary, the volatile composition of coconut meat undergoes a systematic shift from ester-dominated profiles at early maturity to aldehyde-dominated profiles at full maturity. This transition, together with the concurrent changes in lipid and phenolic contents, supports a maturity-based differentiation of coconut meat for specific processing purposes. The selection of raw materials according to the desired flavor direction-fruity and floral for fresh-type products versus roasted and nutty for thermally processed products-can be guided by the volatile profile characteristics of each maturity stage.

## 4. Conclusions

This study demonstrates that maturity is a key determinant of coconut meat quality. As maturation progressed, moisture decreased while fat, protein, soluble sugars, and crude fiber accumulated. Phenolic content declined, but antioxidant activity varied across assays: DPPH scavenging and ferrous ion chelating were highest at CM-8, while ABTS and hydroxyl radical scavenging peaked at CM-12. α-Amylase inhibition was optimal at CM-10. Fatty acids showed transient unsaturation at CM-10 before reverting to a highly saturated profile at CM-12, and volatile compounds shifted from ester-dominated to aldehyde-dominated with maturity. Minerals such as phosphorus, magnesium, zinc, and copper were most abundant at CM-12. These findings provide a scientific basis for stage-specific harvesting and graded utilization, allowing the coconut industry to select raw materials according to desired product attributes. Future research should validate the observed bioactivities through in vivo studies and evaluate consumer acceptance of products derived from different maturity stages.

## Figures and Tables

**Table 1:** Proximate composition of coconut meat with different maturity stages (%).

Sample (%)	CM-8	CM-10	CM-12
Moisture	81.15 ± 1.16 ^a^	76.23 ± 0.76 ^b^	61.73 ± 0.78 ^c^
Ash	0.76 ± 0.04 ^b^	0.96 ± 0.02 ^a^	0.84 ± 0.15 ^ab^
Protein	0.60 ± 0.08 ^c^	0.95 ± 0.07 ^b^	1.29 ± 0.08 ^a^
Fat	8.61 ± 0.12 ^c^	12.49 ± 1.18 ^b^	17.53 ± 0.37 ^a^
Total soluble sugar	2.13 ± 0.11 ^b^	2.26 ± 0.33 ^b^	4.84 ± 0.55 ^a^
Crude fibre	8.85 ± 0.86 ^b^	7.86 ± 0.58 ^b^	13.36 ± 1.89 ^a^

^a–c^ in the same row indicate significant differences (*p* < 0.05).

**Table 2:** Amino acid composition of coconut meat at different maturity stages (mg/g sample).

Amino Acid *	CM-8	CM-10	CM-12
Essential amino acid			
Thr	0.55 ± 0.01 ^c^	0.6 ± 0.01 ^b^	0.99 ± 0.01 ^a^
Val	0.84 ± 0.02 ^c^	0.92 ± 0.01 ^b^	1.7 ± 0.02 ^a^
Met	0.24 ± 0.01 ^c^	0.27 ± 0.01 ^b^	0.55 ± 0.15 ^a^
Ile	0.55 ± 0.01 ^c^	0.62 ± 0.01 ^b^	1.1 ± 0.02 ^a^
Leu	1.05 ± 0.01 ^c^	1.17 ± 0.01 ^b^	2.14 ± 0.02 ^a^
Phe	0.85 ± 0.02 ^c^	0.96 ± 0.01 ^b^	1.52 ± 0.02 ^a^
Lys	0.94 ± 0.01 ^c^	1.02 ± 0.01 ^b^	1.69 ± 0.02 ^a^
His	0.37 ± 0.01 ^c^	0.4 ± 0.01 ^b^	0.78 ± 0.01 ^a^
Total essential amino acid	5.41 ± 0.06 ^c^	5.97 ± 0.03 ^b^	10.46 ± 0.12 ^a^
Non-essential amino acid			
Asp	1.37 ± 0.02 ^c^	1.52 ± 0.01 ^b^	2.82 ± 0.01 ^a^
Ser	0.81 ± 0.02 ^c^	0.9 ± 0.01 ^b^	1.62 ± 0.02 ^a^
Glu	3.08 ± 0.06 ^c^	3.4 ± 0.01 ^b^	6.66 ± 0.03 ^a^
Gly	0.74 ± 0.01 ^c^	0.84 ± 0.01 ^b^	1.56 ± 0.02 ^a^
Ala	1.66 ± 0.03 ^c^	1.85 ± 0.02 ^a^	1.79 ± 0.01 ^b^
Cys	0.14 ± 0.01 ^b^	0.14 ± 0.01 ^b^	0.34 ± 0.01 ^a^
Arg	2.93 ± 0.01 ^c^	3.11 ± 0.01 ^b^	5.3 ± 0.02 ^a^
Tyr	0.39 ± 0.01 ^c^	0.47 ± 0.01 ^b^	0.77 ± 0.02 ^a^
Pro	0.74 ± 0.01 ^c^	0.78 ± 0.01 ^b^	1.33 ± 0.02 ^a^
Total non-essential amino acid	11.86 ± 0.2 ^c^	12.99 ± 0.02 ^b^	22.20 ± 0.01 ^a^
EAA/NEAA	0.46 ± 0.01 ^a^	0.46 ± 0.01 ^a^	0.47 ± 0.01 ^a^
EAA/TAA (%)	31.25 ± 0.05 ^a^	31.42 ± 0.08 ^a^	32.06 ± 0.24 ^a^

* Thr: Threonine; Val: Valine; Met: Methionine; Ile: Isoleucine; Leu: Leucine; Phe: Phenylalanine; Lys: Lysine; His: Histidine; Asp: Aspartic acid; Ser: Serine; Glu: Glutamic acid; Gly: Glycine; Ala: Alanine; Cys: Cysteine; Arg: Arginine; Tyr: Tyrosine; Pro: Proline; EAA: Essential amino acid; NEAA: Non-essential amino acid; TAA: Total amino acid. Values marked with different lowercase letters ^(a–c)^ within the same row are significantly different at *p* < 0.05.

**Table 3:** Fatty acid composition of coconut meat at different maturity stages (% of total fatty acids).

Fatty Acids *	CM-8 (%)	CM-10 (%)	CM-12 (%)
Caproic acid (C_6:0_)	0.25 ± 0.01 ^b^	0.24 ± 0.02 ^b^	0.34 ± 0.03 ^a^
Caprylic acid (C_8:0_)	4.28 ± 0.01 ^b^	3.34 ± 0.08 ^c^	4.77 ± 0.03 ^a^
Capric acid (C_10:0_)	4.56 ± 0.01 ^a^	3.36 ± 0.02 ^c^	4.49 ± 0.04 ^b^
Lauric acid (C_12:0_)	51.22 ± 0.12 ^a^	41.16 ± 0.15 ^c^	46.87 ± 0.17 ^b^
Myristic acid (C_14:0_)	22.41 ± 0.04 ^a^	20.8 ± 0.02 ^c^	21.56 ± 0.03 ^b^
Palmitic acid (C_16:0_)	11.77 ± 0.19 ^b^	12.41 ± 0.28 ^a^	10.7 ± 0.05 ^c^
Stearic acid (C_18:0_)	4.26 ± 0.03 ^b^	3.88 ± 0.21 ^c^	4.54 ± 0.08 ^a^
Oleic acid (C_18:1n9c_)	-	13.27 ± 0.26 ^a^	5.66 ± 0.04 ^b^
Linoleic acid (C_18:2n6c_)	1.25 ± 0.01 ^b^	1.54 ± 0.04 ^a^	1.19 ± 0.03 ^c^
SFA	98.75 ± 0.01 ^a^	85.19 ± 0.27 ^c^	93.27 ± 0.17 ^b^
UFA	1.25 ± 0.01 ^c^	14.81 ± 0.27 ^a^	6.85 ± 0.03 ^b^
MUFA	-	13.27 ± 0.26 ^a^	5.66 ± 0.04 ^b^
PUFA	1.25 ± 0.01 ^b^	1.54 ± 0.04 ^a^	1.19 ± 0.03 ^c^

* SFA, UFA, MUFA, and PUFA represent the percentage of saturated fatty acids, total unsaturated fatty acids, monounsaturated fatty acids, and polyunsaturated fatty acids, respectively; “-” in the table indicates that the fatty acid was not detected. Values marked with different lowercase letters ^(a–c)^ within the same row are significantly different at *p* < 0.05.

**Table 4:** Mineral element contents of coconut meat at different maturity stages (mg/kg) and recommended nutrient intake (RNI) reference values.

Mineral Elements	CM-8	CM-10	CM-12	RNI (mg/d)
Macro-minerals (mg/kg)				
Potassium	5097.32 ± 75.81 ^a^	4277.12 ± 82.55 ^c^	4753.91 ± 59.16 ^b^	350–2200
Phosphorus	707.29 ± 33 ^b^	617.45 ± 16.11 ^c^	1049.08 ± 11.82 ^a^	100 *–720
Sodium	49.3 ± 0.27 ^b^	210.84 ± 4.51 ^a^	42.23 ± 0.41 ^c^	170–1600
Magnesium	482.42 ± 15.25 ^b^	445.9 ± 15.56 ^c^	617.79 ± 6.91 ^a^	20 *–330
Calcium	123.79 ± 9.31 ^a^	125.06 ± 6.08 ^a^	104.12 ± 0.38 ^b^	200 *–1200
Micro-minerals (mg/kg)				
Iron	18.93 ± 1.54 ^ab^	10.53 ± 2.32 ^b^	20.44 ± 0.68 ^a^	0.3 *–20
Zinc	2.88 ± 0.33 ^b^	2.45 ± 0.04 ^c^	5.51 ± 0.04 ^a^	2.0 *–12.5
Manganese	8.66 ± 0.67 ^b^	9.84 ± 0.42 ^a^	10.64 ± 0.15 ^a^	0.01 *–4.5 *
Copper	1.77 ± 0.07 ^b^	1.18 ± 0.01 ^c^	4.21 ± 0.07 ^a^	0.3 *–0.8
Tin	0.79 ± 0.1 ^a^	0.65 ± 0.06 ^a^	0.75 ± 0.09 ^a^	-
Nickel	0.47 ± 0.02 ^a^	0.39 ± 0.01 ^b^	0.35 ± 0.01 ^c^	-
Other minerals (mg/kg)				
Aluminum	1.0 ± 0.15 ^a^	0.73 ± 0.03 ^b^	0.86 ± 0.05 ^ab^	-
Boron	1.03 ± 0.14 ^a^	0.81 ± 0.02 ^b^	0.99 ± 0.06 ^a^	-
Strontium	0.2 ± 0.02 ^b^	0.42 ± 0.01 ^a^	0.13 ± 0.01 ^c^	-

RNI denotes the recommended nutrient intake, and asterisk (*) indicates adequate intake (AI); Values marked with different lowercase letters ^(a–c)^ within the same row are significantly different at *p* < 0.05; “-” indicates that no RNI value has been established.

**Table 5:** Identified volatile compounds and their contents in coconut meat at different maturity stages.

Number	Compound Name	CAS#	Retention Index	Retention Time (s)	Drift Time (ms)	Content (μg/Kg) ^a^		
						CM-8	CM-10	CM-12
	Ketones (2)							
1	2-Heptanone	C110430	889.3	381.829	1.26396	6.02 ± 0.68 ^a^	4.07 ± 0.12 ^b^	5.18 ± 0.39 ^a^
2	3-Hydroxybutan-2-one	C513860	712.6	195.362	1.32751	25.90 ± 3.31 ^a^	14.96 ± 1.20 ^b^	1.95 ± 0.21 ^c^
	Aldehydes (4)							
3	4-Methylbenzaldehyde	C104870	1073.7	720.539	1.1948	1.47 ± 0.37 ^a^	1.27 ± 0.09 ^a^	1.31 ± 0.10 ^a^
4	Nonanal	C124196	1106.3	790.374	1.49178	2.81 ± 0.29 ^ab^	2.53 ± 0.06 ^b^	3.18 ± 0.38 ^a^
5	Heptanal	C111717	903	402.542	1.35482	0.88 ± 0.10 ^b^	0.80 ± 0.07 ^b^	1.60 ± 0.08 ^a^
6	Propanal	C123386	469.9	100.18	1.15237	84.67 ± 14.44 ^a^	61.20 ± 3.79 ^b^	22.24 ± 12.92 ^c^
	Esters (10)							
7	Hexyl butanoate	C2639636	1185	1002.887	1.48513	17.57 ± 1.66 ^b^	20.86 ± 1.10 ^a^	9.50 ± 0.36 ^c^
8	Methyl heptanoate ^M^	C106730	1007.4	597.231	1.33876	13.54 ± 1.88 ^a^	13.46 ± 0.57 ^a^	3.31 ± 0.16 ^b^
9	Methyl heptanoate ^D^	C106730	1009	600.1	1.78649	4.89 ± 0.95 ^a^	4.82 ± 0.15 ^a^	2.19 ± 0.14 ^b^
10	Acetic acid butyl ester	C123864	813.4	285.834	1.22898	5.94 ± 1.38 ^a^	4.90 ± 1.28 ^a^	3.66 ± 1.06 ^a^
11	Pentyl formate	C638493	810.8	283.053	1.29595	0.92 ± 0.15 ^a^	0.79 ± 0.20 ^a^	0.84 ± 0.27 ^a^
12	Methyl 3-methylbutanoate	C556241	778.6	250.381	1.20245	3.50 ± 0.73 ^a^	2.71 ± 0.33 ^a^	1.43 ± 0.37 ^b^
13	*n-*Propyl acetate	C109604	708.8	192.629	1.16039	54.00 ± 16.39 ^a^	55.14 ± 5.62 ^a^	13.36 ± 4.21 ^b^
14	Butyl formate	C592847	736.3	213.584	1.50489	6.00 ± 0.63 ^a^	4.07 ± 0.45 ^b^	1.25 ± 0.10 ^c^
15	Acetic acid ethyl ester ^D^	C141786	607.9	144.246	1.33521	2.56 ± 1.81 ^a^	3.47 ± 2.13 ^a^	0.85 ± 0.18 ^a^
16	Acetic acid ethyl ester ^M^	C141786	607.9	144.246	1.11485	20.26 ± 8.91 ^a^	18.06 ± 7.51 ^a^	6.42 ± 3.59 ^a^
	Alcohols (8)							
17	2-Octanol	C123966	1007.8	597.948	1.44847	7.91 ± 0.95 ^a^	8.73 ± 0.36 ^a^	2.61 ± 0.18 ^b^
18	2-Ethyl-1-hexanol	C104767	1047	668.125	1.42928	4.54 ± 0.62 ^a^	3.27 ± 0.10 ^b^	3.34 ± 0.17 ^b^
19	*n-*Hexanol	C111273	872.1	357.545	1.3277	3.00 ± 0.60 ^b^	4.23 ± 0.36 ^a^	3.41 ± 0.39 ^ab^
20	Heptan-2-ol	C543497	890.3	383.258	1.36269	0.99 ± 0.10 ^b^	1.03 ± 0.16 ^b^	2.20 ± 0.12 ^a^
21	Pentan-2-ol	C6032297	713.2	195.818	1.21268	39.98 ± 4.10 ^a^	30.57 ± 0.28 ^b^	10.95 ± 1.54 ^c^
22	1-Butanol ^M^	C71363	654.6	163.175	1.1932	17.20 ± 3.21 ^b^	26.04 ± 2.31 ^a^	21.88 ± 1.62 ^ab^
23	1-Butanol ^D^	C71363	655.7	163.666	1.39621	3.56 ± 1.27 ^b^	12.52 ± 2.52 ^a^	3.45 ± 2.20 ^b^
24	2-Propanol	C67630	459.2	97.38	1.09245	145.81 ± 25.93 ^a^	122.54 ± 4.62 ^a^	78.63 ± 16.11 ^c^
	Acids (6)							
25	3-Methyl valeric acid	C105431	963.8	509.836	1.27794	2.08 ± 0.25 ^a^	1.95 ± 0.04 ^a^	1.93 ± 0.21 ^a^
26	Hexanoic acid	C142621	996.9	579.673	1.30871	1.87 ± 0.15 ^a^	1.76 ± 0.27 ^a^	2.14 ± 0.94 ^a^
27	Butanoic acid ^M^	C107926	815.9	288.614	1.16581	11.80 ± 4.69 ^a^	10.06 ± 3.23 ^a^	7.59 ± 4.51 ^a^
28	Butanoic acid ^D^	C107926	809.6	281.663	1.35913	1.68 ± 0.45 ^a^	1.58 ± 0.36 ^a^	1.87 ± 0.39 ^a^
29	Propanoic acid ^M^	C79094	713.7	196.171	1.09734	10.93 ± 2.72 ^a^	13.67 ± 2.63 ^a^	9.90 ± 2.79 ^a^
30	Propanoic acid ^D^	C79094	714.1	196.515	1.27034	6.58 ± 0.44 ^a^	5.03 ± 0.31 ^b^	1.63 ± 0.33 ^c^
	Others (1)							
31	Allyl propyl sulfide	C27817670	869.1	353.517	1.399	2.47 ± 0.74 ^a^	2.97 ± 0.26 ^a^	1.21 ± 0.15 ^b^

^a^ Contents are expressed as μg/kg on a fresh weight basis. ^M^ and ^D^ denote the monomeric and dimeric forms of the compound detected in the GC-IMS system, respectively. CAS# refers to the Chemical Abstracts Service registry number assigned to each chemical substance by the American Chemical Society. Different lowercase superscript letters ^(a–c)^ within the same row indicate significant differences at *p* < 0.05.

## Data Availability

All data presented in this study are available on request from the corresponding author. The data are not uploaded in publicly accessible databases.

## References

[1] Djafar F., Maghfirah N.A., Syamsuddin Y., Faisal M., Supardan M.D. (2026). Comparative evaluation of sun, oven, and microwave drying of grated coconut (*Cocos nucifera* L.): Linking drying kinetics, microstructure, and oil recovery. Appl. Food Res..

[2] Xiong L., Jiang G., Wei J., Xu Y., Feng W., Li J. (2026). Dietary supplementation of coconut meat modulates growth performance, nutritional composition, and internal regulation in Chinese mitten crab (*Eriocheir sinensis*). Front. Nutr..

[3] Shi Y., Xun L., Li Y., Wu G. (2026). Influence of coconut meat powder on the quality and antioxidant activity of rice wine. Food Hum..

[4] Sahari Y., Anuar M.S., Mohd Nor M.Z., Abdul Ghani N.H., Mohd Tahir S. (2023). Progress, trends and development of drying studies on coconut kernel products: A review. Pertanika J. Sci. Technol..

[5] Liu X.Y., Yang D.W., Liu W.T., Kan J.T., Zhang Y.F. (2024). Effect of Dry processing of coconut oil on the structure and physicochemical properties of coconut isolate proteins. Foods.

[6] Karim A.A., Nassima C., Tristan R. (2022). Analysis of individual anthocyanins, flavanols, flavonols and other polyphenols in *Pistacia lentiscus* L. fruits during ripening. J. Food Compost. Anal..

[7] Goldberg T., Agra H., Ben-Arie R. (2021). Quality of ‘Hayward’ kiwifruit in prolonged cold storage as affected by the stage of maturity at harvest. Horticulturae.

[8] Shayanthavi S., Kapilan R., Wickramasinghe I. (2024). Comprehensive analysis of physicochemical, nutritional, and antioxidant properties of various forms and varieties of tender coconut (*Cocos nucifera* L.) water in Northern Sri Lanka. Food Chem. Adv..

[9] Bhanu P.R.N., Thivya T., Sinija V.R. (2026). Impact of pulsed electric field, high-pressure processing, combined, and thermal treatments on the quality of tender coconut water during refrigerated preservation. Food Bioprod. Process..

[10] Jiang R., Xue D., Chen Y., Lv X., Ni L., Liu Z. (2025). Comparative study of the fatty acid and phenolic profiles of tender and mature coconut for coconut milk production. Foods.

[11] Rajesh M.K., Budhwar R., Shukla R., Oraon P.K., Goel S., Paul B. (2024). Chromosome scale genome assembly and annotation of coconut cultivar Chowghat Green Dwarf. Sci. Rep..

[12] Subhaktian D., Evizal R., Novpriansyah H., Karyanto A. (2025). The effect of salicylic acid and naphthalene acetic acid on reducing button shedding in dwarf coconut (*Cocos nucifera* L.). Proceedings of the IOP Conference Series: Earth and Environmental Science.

[13] Zhang Y.F., Kan J.T., Liu X.Y., Song F., Zhu K.X., Li N. (2024). Chemical components, nutritional value, volatile organic compounds and biological activities in vitro of coconut (*Cocos nucifera* L.) water with different maturities. Foods.

[14] Namitha M.Y., Ravikumar Patil H.S., Kiran Kuma H.B. (2025). Physico-chemical analysis of foxtail millet implications for nutrition and value addition in food products. Asian J. Res. Biochem..

[15] Wang K., Zeng J., Chen Y., Wang Y. (2026). Comparative analysis of total phenols, total flavonoids, antioxidant capacity, and xanthine oxidase inhibition in six types of tea. Foods.

[16] Lin Y., Ge X., Mao M., Liu J., Jin Z., Wang Y. (2026). Structural characterization and neuroprotective effect of coix seed polysaccharides. Food Biosci..

[17] Rohman M.A., Rahman M.N., Halim M.A., Sohany M., Mahomud M.S. (2026). Optimization of ultrasound-assisted extraction conditions for phenolic content and antioxidant activity in green mango peel using response surface methodology. Food Sci. Nutr..

[18] Zhang Y., Duan X., Zhuang Y. (2012). Purification and characterization of novel antioxidant peptides from enzymatic hydrolysates of tilapia (*Oreochromis niloticus*) skin gelatin. Peptides.

[19] Hu T.Y., Tu C.Z., Liu X.G. (2026). Enzymatic hydrolysis affected the antioxidant activity and traceability identification of porcine collagen peptides. Food Chem..

[20] Coronado S.R., Sánchez S.F.N., Cruz G.N. (2026). Relationship between antioxidant properties and α-amylase and α-glucosidase inhibitory capacity of *Justicia spicigera* leaf extracts. Phytomed. Plus.

[21] Tas I., Akcura S., Kaplan M., Jagosz B., Atılgan A., Kocięcka J. (2024). The effect of drip irrigation and nitrogen levels on the oil and fatty acid composition of sesame and its economic analysis. Agronomy.

[22] Yang J., Sun P., He Y. (2026). Characteristic of mineral elements and volatile profiles in sixteen hybrid table grape cultivars (*Vitis labrusca × Vitis vinifera*) from Shanghai, China. J. Food. Compost. Anal..

[23] Frangipane M.T., Costantini L., Garzoli S., Merendino N., Massantini R. (2026). Sensory profiles, volatile compounds and antioxidant activity of organically grown almonds (*Prunus dulcis* Mill. DA Webb). Agriculture.

[24] Gómez-Tah R., Islas-Flores I., Félix J.W., Granados-Alegría M.I., Tzec-Simá M., Guerrero-Analco J.A. (2023). Untargeted metabolomics analysis of liquid endosperm of *Cocos nucifera* L. at three stages of maturation evidenced differences in metabolic regulation. Horticulturae.

[25] Félix J.W., Granados-Alegría M.I., Gómez-Tah R., Tzec-Simá M., Ruíz-May E., Canto-Canché B. (2023). Proteome landscape during ripening of solid endosperm from two different coconut cultivars reveals contrasting carbohydrate and fatty acid metabolic pathway modulation. Int. J. Mol. Sci..

[26] Sulaeman A., Nasution Z., Setiawan B., Azra J.M. (2021). Effects of variety and maturity stage of coconut on physicochemical and sensory characteristics of powdered coconut drink. Foods Raw Mater..

[27] Manivannan A., Bhardwaj R., Padmanabhan S., Suneja P., Hebbar K.B., Kanade S.R. (2018). Biochemical and nutritional characterization of coconut (*Cocos nucifera* L.) haustorium. Food Chem..

[28] Buyukkurt O.K., Guclu G., Kelebek H., Selli S. (2025). Comparative elucidation of the phenolic profile and antioxidant and antimicrobial activities of unripe and ripe *Pistacia lentiscus* L. fruits and their oils as affected by ripening. J. Sci. Food Agric..

[29] Hnialum L., Chatli M.K. (2026). Antioxidant and antimicrobial potential of peptidic hydrolysates from duck egg proteins. Nutr. Food Sci..

[30] Faleye B.R., Flores-Gallegos A.C., Ascacio-Valdés J.A., Orozco Sifuentes M.M., Silva-Belmares S.Y., Nery-Flores S.D. (2026). *Ferocactus pilosus* buds: Nutritional significance, techno-functional properties, antioxidant and antibacterial potential. Biocatal. Agric. Biotechnol..

[31] Riaz M., Khalid R., Afzal M., Anjum F., Fatima H., Zia S. (2023). Phytobioactive compounds as therapeutic agents for human diseases: A review. Food Sci. Nutr..

[32] Fierro O., Siano F., Puppo M.C., Mamone G., Picariello G. (2026). Flavonoid C-glycosides in *Fabaceae* seeds: From structural diversity and functional significance to human health benefits. Food Chem..

[33] Yang J., Li H., Wang X., Zhang C., Feng G., Peng X. (2021). Inhibition mechanism of α-amylase/α-glucosidase by silibinin, its synergism with acarbose, and the effect of milk proteins. J. Agric. Food Chem..

[34] Tulashie S.K., Amenakpor J., Atisey S., Odai R., Akpari E.E.A. (2022). Production of coconut milk: A sustainable alternative plant-based milk. Case Stud. Chem. Environ. Eng..

[35] Abdul H.S., Iqbal Zafar M. (2011). Chemical composition of meat (kernel) and nut water of major coconut (*Cocos nucifera* L.) cultivars at coastal area of pakistan. Pak. J. Bot..

[36] Adeyeye E.I. (2004). The chemical composition of liquid and solid endosperm of ripe coconut. Orient. J. Chem..

[37] Peterson L.R., Culotta C.V. (2026). From hemocuprein to CSRP: The many faces of Cu/Zn superoxide dismutase. Metallomics.

[38] Qian B., Zhu X., Su C., Li H., Wu Q., Liu C. (2026). Mechanism of water-enhanced volatile aldehyde release in oil fumes from thermal oxidation of oleic acid: Insights from synchrotron radiation photoionization and gas chromatography-mass spectrometry. Molecules.

[39] Niu Y., Fu X., Zhou Z., Qiu M., Zhou B., Fan J. (2026). Dynamic evolution of characteristic flavor molecules in Yunnan arabica coffee during roasting revealed with a multi-omics framework. Food Chem..

